# A genome-wide screen in human embryonic stem cells reveals novel sites of allele-specific histone modification associated with known disease loci

**DOI:** 10.1186/1756-8935-5-6

**Published:** 2012-05-19

**Authors:** James G D Prendergast, Pin Tong, David C Hay, Susan M Farrington, Colin A M Semple

**Affiliations:** 1 MRC Human Genetics Unit, MRC Institute of Genetics and Molecular Medicine, University of Edinburgh, Western General Hospital, Crewe Road, , Edinburgh, EH4 2XU, UK; 2 UCD Conway Institute for Biomolecular and Biomedical Research, Dublin, Ireland; 3 MRC Centre for Regenerative Medicine, University of Edinburgh, 49 Little France Crescent, Edinburgh, EH16 4SB, UK

## Abstract

**Background:**

Chromatin structure at a given site can differ between chromosome copies in a cell, and such imbalances in chromatin structure have been shown to be important in understanding the molecular mechanisms controlling several disease loci. Human genetic variation, DNA methylation, and disease have been intensely studied, uncovering many sites of allele-specific DNA methylation (ASM). However, little is known about the genome-wide occurrence of sites of allele-specific histone modification (ASHM) and their relationship to human disease. The aim of this study was to investigate the extent and characteristics of sites of ASHM in human embryonic stem cells (hESCs).

**Results:**

Using a statistically rigorous protocol, we investigated the genomic distribution of ASHM in hESCs, and their relationship to sites of allele-specific expression (ASE) and DNA methylation. We found that, although they were rare, sites of ASHM were substantially enriched at loci displaying ASE. Many were also found at known imprinted regions, hence sites of ASHM are likely to be better markers of imprinted regions than sites of ASM. We also found that sites of ASHM and ASE in hESCs colocalize at risk loci for developmental syndromes mediated by deletions, providing insights into the etiology of these disorders.

**Conclusion:**

These results demonstrate the potential importance of ASHM patterns in the interpretation of disease loci, and the protocol described provides a basis for similar studies of ASHM in other cell types to further our understanding of human disease susceptibility.

## Background

In genome-wide studies of human chromatin, chromatin states are often assumed to be the same on both copies of a chromosome. However, an increasing number of studies has shown that a surprising level of heterogeneity can in fact exist between the chromatin states of different alleles. A recent study of allele-specific methylation (ASM) of DNA in various pluripotent and adult cell lines estimated that 23 to 37% of heterozygous single-nucleotide polymorphisms (SNPs) displayed differing levels of methylation between alleles, with most ASM sites attributable to heterozygote polymorphisms disrupting the guanine nucleotide in CpG motifs [1].

Although DNA methylation is the best-characterized mechanism [2], it is not the only level of chromatin that has been shown to exhibit genome-wide allele-specific patterns in humans. For example, the nuclease DNaseI has been shown to cleave at sites of open chromatin and to preferentially target regulatory elements such as promoters and enhancers. Investigation of allele-specific patterns of DNaseI hypersensitivity has shown that 7% of DNaseI sites display allelic imbalances in sensitivity [3].

Despite the crucial roles of histone modifications in various biological processes, there has been no systematic investigation to date of the extent of human ASHM genome-wide. At present, only a handful of sites have been found in the human genome, where histone modifications are preferentially or exclusively associated with a specific SNP allele [4,5]. The sites discovered in mice, in which allele-specific histone modification (ASHM) has been studied more extensively, have generally been found around known imprinted regions [6-9]. Although ASHM is therefore known to occur at particular imprinted loci in mammals, often in conjunction with allele-specific DNA methylation [8,10], the degree to which it is associated with imprinted loci genome-wide, and the extent of the overlap between ASHM and ASM across the human genome remains unknown. Similarly, the relative effect of ASM and ASHM on gene-expression patterns across the genome is poorly understood.

Allele-specific patterns of other forms of chromatin are associated with various disorders such as diabetes [11-13] and despite being poorly characterized to date, patterns of ASHM are likely to be important in understanding the incidence and progression of various diseases [14]. Histone modifications are associated with a variety of biological processes, most notably the regulation of gene expression, and patterns of histone modification specific to the parent of origin of the corresponding chromosome, have already been associated with disorders such as Prader-Willi syndrome (PWS) [15]. More generally, if sites of ASHM are associated with the expression of neighboring genes, it might be expected that such genes would be unusually sensitive to further perturbations in their function. For example, if an ASHM variant is associated with a loss of expression of one allele of a gene, then expression of this gene will be particularly susceptible to disruption via mutations of the second allele.

The discovery of regions of allele-specific chromatin structure is consequently important for the interpretation of variants and their phenotypic effects in genome-wide association studies (GWAS) [16,17]. The targeted investigation of associations between disease and SNPs at known imprinted regions has been shown to give stronger SNP-disease associations when the chromatin status of each allele is taken into account [18]. Outside imprinted regions, the discovery of sites at which chromatin state is associated with a particular allele or haplotype will assist the interpretation of GWAS results, and conceivably the detection of causal variants.

In this study, we performed the first systematic survey of allele-specific patterns of histone-modification patterns in the human genome, by exploiting the unparalleled, large volume of chromatin, expression, and genotyping data now available for a widely used human embryonic stem cell (hESC) line. We documented the extent of ASHM across 23 well-studied histone modifications in the H1 cell line, and found that ASHM is present throughout the human genome and is detectable outside known imprinted regions. Furthermore, ASHM patterns seem to involve several relatively common allelic imbalances, preferentially involving certain modifications. Through the use of matched DNA methylation data, we also investigated the relationship between ASM and ASHM and their relative enrichments around known imprinted loci, and identified ASHM is more specific in detecting imprinted regions. Both DNA methylation and histone-modification patterns are known to be associated with altered gene expression, but for the first time we quantified their relative contribution to sites of allele-specific gene expression. We compared the locations of sites of ASHM identified in the H1 cell line to those found in the fetal lung fibroblast cell line, IMR90, and finally, we investigated the colocalization of sites of ASHM in embryonic stem cells and known disease loci associated with human developmental disorders [19].

## Results and discussion

### Distinct allele-specific histone imbalances across the human genome

To identify sites of allele-specific DNA modification and expression in the H1 hESC line, the locations of heterozygous polymorphisms were first identified. Although H1 has previously been genotyped, providing the location of 153,718 heterozygous SNPs [20], each individual is expected to carry more than 1.6 million heterozygous SNPs [21]. This H1 genotyping-array data was therefore used in conjunction with HapMap [22] and 1000 genomes [21] haplotypes to impute the locations of a further 1,572,866 putative heterozygote sites (IMPUTE [23] probability of being a heterozygote > 0.5). The resulting set of imputed heterozygotes was likely to contain false positives, and so all putative heterozygotes were validated using sequencing data (see Methods).

We successful aligned 1.4 billion reads from a variety of H1 chromatin immunoprecipitation (ChIP)-sequencing datasets [24,25] (corresponding to a total of 23 different histone modifications; see Additional file 1) to the human reference genome. Known heterozygous sites, at which two or more histone modifications displayed significantly different proportions of reads carrying each allele, were identified. To ensure that any imbalance detected was not simply a result of a shift in nucleosome positioning between alleles (which would lead to similar patterns of allelic imbalances for all modifications at a position), we considered only sites displaying evidence of different spectrums of histone modifications on each allele to be sites of ASHM (rather than making an assessment of allelic imbalance for each modification in isolation; Table 1). It should be noted that this is currently an unprecedented and unique dataset for the comprehensive identification of ASHM, offering the first impression of the frequency and patterns of this phenomenon across the entire human genome.

**Table 1:** **Modifications displaying allelic imbalance in each of the clusters shown in Figure**1A

	**Modifications preferentially associated with:**	
**Cluster**	**Allele A**	**Allele B**
1	H3K4me3	–
2	H3K4me3	H3K27me3
3	H3K27me3	–
4	H3K36me3	–
5	H3K9me3	–
6	H3K9me3	H3K36me3

**Figure 1:**
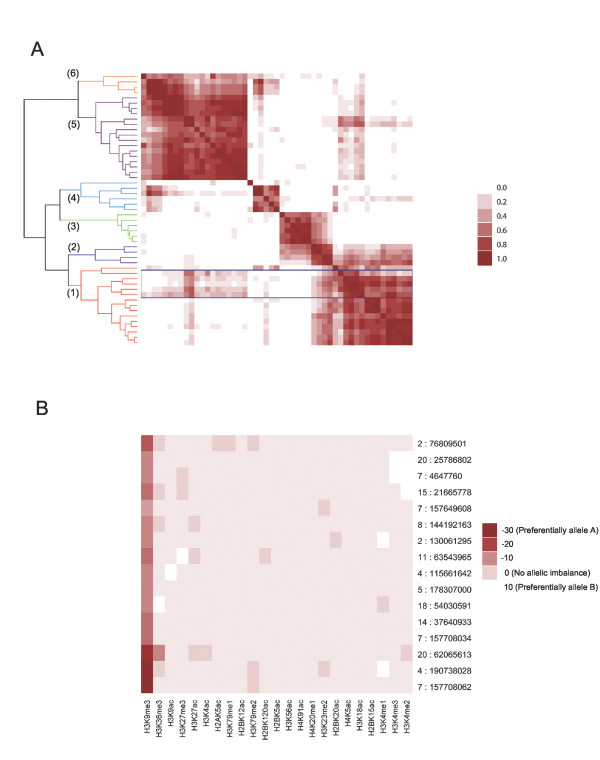
**Clustering of significant sites of allele-specific histone modification in the H1 cell line by patterns of allele-specific modification. (A)** All 51 significant sites clustered according to the allelic imbalance seen for each examined histone modification. The scale corresponds to the squared correlation of the observed allelic imbalances for each modification (as measured by the log of the respective binomial *P*-value) between corresponding pairs of sites. Sites showing the strongest evidence of being largely driven by H3K4me3 preferentially on one allele and H3K9me3 on the other are highlighted by the blue box (chr4:89004339, chr4:89004397, chr4:89004505, chr7:23729054, chr15:22751085). **(B)** Allelic imbalance for each histone modification at the sites forming cluster 5. Most modifications displayed no consistent evidence of allelic imbalance at these positions; however, H3K9me3 reads were found to preferentially contain allele ‘A’ at these sites (for this plot the allele chosen to be the ‘A’ allele at each site was the allele found more often in the H3K9me3 reads at each position).

In total, 51 sites (see Additional file 2) displaying evidence of ASHM with *P* < 1.0 × 10^−7^ remained after stringent filtering (see Methods). Clustering these sites according to their allelic imbalance for each modification highlighted that there were at least six different patterns of ASHM at these positions (Figure 1A). Some ASHM sites seemed to be driven by imbalances on a single copy of a chromosome, with the sites that formed cluster 5 displaying consistent evidence of ASHM for the H3K9me3 marker (Table 1, Figure 1B);one allele at these loci being preferentially associated with nucleosomes carrying the H3K9me3 modification, with no modification preferentially associated with the second allele. However, sites at which different modifications are favored on each allele were also identified. For example, sites in cluster 2 generally carry H3K27me3-modified (a marker of transcriptional repression) nucleosomes on one allele and H3K4me3-modified (associated with transcriptionally active regions) nucleosomes on the other. Although the ability to detect certain patterns of allelic imbalances will depend on the coverage of the different modifications in this study, these results highlight that a spectrum of different forms of ASHM are present in the human genome.

### Allele-specific histone modifications are preferentially linked to sites of allele-specific expression

**Figure 2:**
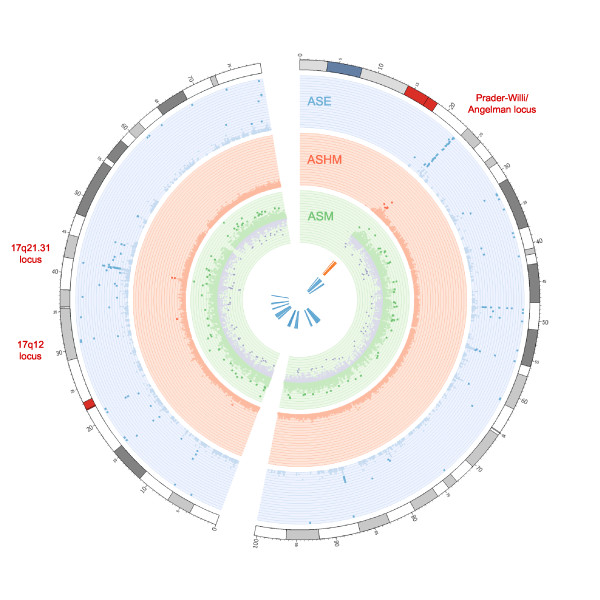
**Patterns of allele-specific expression (ASE), histone modification (ASHM), and methylation (ASM) along chromosomes 15 (right) and 17 (left).** Sites of ASE are plotted in the outer blue circle, ASHM in the middle red circle, and ASM in the inner green plot. Significant sites are shaded darker in each case. Points correspond to –log( *P*) values in each plot except for ASM sites on the negative reference strand, where log( *P*) is plotted (purple points in the inner green plot). Known imprinted loci and indel regions associated with developmental disorders are indicated by orange and blue boxes, respectively, at the centre of the plot. Names of regions associated with developmental disorders within 250 kb of a site of ASHM are shown in red along with positions (in Mb) around the outside of the figure [28].

Of the various biological processes associated with histone modifications, the best characterized is the association of these modifications with regulation of gene expression. For example, the H3K27me3 modification has been associated with repression and the H3K4me3 marker with activation of gene expression [26], and therefore it is likely that allele-specific modification patterns such as those we found in cluster 2 (Table 1) may be reflected in the expression patterns of nearby genes. To investigate the potential effect of ASHM on gene expression, we combined various RNA-sequencing datasets derived from the H1 cell line, and analyzed them for evidence of ASE at genes in close proximity to the sites of ASHM. There was clear evidence of the colocalization of both ASHM and gene expression at many locations in the genome (Figure 2). In total, 19.6% of ASHM sites were within 10 kb of a heterozygote site displaying evidence of ASE (see Additional file 3), compared with 4% of all high-coverage (>35*x*: the minimum coverage of the 51 sites of ASHM; see Methods) heterozygote sites ( *P* = 1.2 × 10^−7^, χ² test). There was also some evidence for enrichment of allele-specific *Pol*II (ASP) sites around locations of ASHM, with five sites of ASHM being within 10 kb of a site of allele-specific *Pol*II binding (see Additional file 3); 9.8% of sites of ASHM versus 0.17% of all high-coverage sites were within the same distance of a location of ASP (*P* = 3.4 × 10^−8^, Fisher’s exact test). Given that many genes did not contain heterozygous sites or possess sufficient RNA-sequencing coverage for ASE to be determined, it is likely that even more ASHM sites than could be detected in this study are in fact in close proximity to genes displaying allele-specific expression. In addition, it is possible that some ASHM sites affect chromatin compaction and long-range interactions, with delayed or more subtle effects on transcription, as has been seen for asthma-associated variants conferring allele-specific chromatin states [27].

### Sites of allele-specific histone modification are more enriched at imprinted regions than are sites of allele-specific methylation

Patterns of allele-specific inactivation of gene expression can sometimes occur, through a process known as imprinting. The imprinting of regions, by which maternal and paternal copies of a gene display different expression levels, has largely been attributed to altered DNA methylation states, with ASM of nearby CpG islands leading to different levels of gene expression from maternally and paternally derived chromosomes [29]. However, there is growing evidence from targeted studies that sites of ASHM may also be acting to modulate the levels of methylation at a locus [30]. To test whether ASHM was preferentially associated with imprinted regions, we compared their positions with the locations of known imprinted genes from the Imprinted Gene Catalogue [31]. A number of the ASHM loci were found to be in close proximity to regions previously shown to be imprinted, with 9 (17.6%) of the 51 most significant ASHM sites being within 10 kb of a known imprinted gene (corresponding to 5 distinct imprinted loci; see Additional file 3). Given that only 0.6% of all high-coverage sites were within 10 kb of a known imprinted locus, this is a substantial (29.3-fold), and highly significant enrichment of ASHM sites around imprinted regions (*P* < 2.2 × 10^−16^, χ² test). This observation is consistent with studies of mouse embryonic fibroblasts, which also found ASHM to be associated with imprinted regions [7,8]. Although many sites of histone-modification imbalances between chromosomes at imprinted regions probably do not overlap a heterozygote SNP and so would not be detected as ASHM, these results illustrate that ASHM is substantially enriched around imprinted regions.

**Figure 3:**
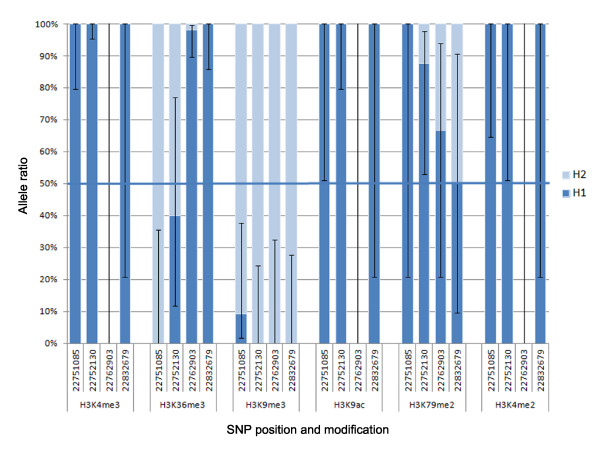
**Allele ratio of the six histone modifications displaying the highest total coverage at four phased heterozygote sites at the Prader-Willi locus.** Sites without a corresponding bar had no reads for the corresponding histone-modification map to that position. The 95% confidence intervals of the allele ratios are shown.

This result suggests that the location of ASHM could be used to prioritize regions that are potentially imprinted. The most significant site of ASHM detected in our study mapped to the well-characterized imprinted locus on 15q11-13. In total, four sites of ASHM were detected in this region, at two of which H3K9me3 was preferentially associated with one chromosome at this locus, and H3K4me3 preferentially associated with the other (Figure 3). This is consistent with previous studies in the mouse, which have shown that overlapping sites of H3K4me3 and H3K9me3 are strongly associated with imprinting control regions [32]. Interestingly, the third, fifth, and sixth most significant sites of ASHM (all mapping to the same locus on chromosome 4) also showed similar evidence of H3K4me3 on one allele and H3K9me3 on the other, and clustered with one of the sites of ASHM at the imprinted region on chromosome 15 (Figure 1A). Although, as far as we are aware, this region is not currently known to be imprinted, the location also displayed allele-specific *Pol*II binding in close proximity, as also observed at the chromosome 15 imprinted locus. This region is consequently a strong candidate for follow-up studies investigating potential imprinting at this locus. Imprinting at this locus would be particularly interesting, given that variants in this region have previously been associated with bone-mineral density in GWAS [33], suggesting that taking into account the parent of origin of alleles at this locus might increase the strength of the observed associations [18].

Although it is not possible to determine whether ASHM is controlling the imprinting at known imprinted loci, or whether it is simply a consequence, the enrichment of ASHM around known imprinted loci is substantial. To compare this enrichment to the extent of enrichment of ASM at imprinted loci, we aligned Bisulfite sequencing reads derived from the H1 cell line [34] to the reference genome using the software Bismark [35]. Sites of ASM were identified in a strand-specific manner by identifying allelic imbalances at base positions of at least partial methylation. Examination of the distribution of these ASM sites in the genome highlighted that, as reported by other studies [1], an overwhelming majority were adjacent to polymorphisms, as most allele-specific methylation sites are a result of the guanine at CpG sites being polymorphic, leading to the disruption of the CpG dinucleotide. Using our strict, Bonferroni-corrected *P*-value threshold of 1 × 10^−8^, only 38 out of the 7,569 significant sites of ASM were not located directly adjacent to polymorphisms in the H1 cell line, with an imputed heterozygote probability of greater or equal to 0.5. It is possible that at least some of the remaining 38 SNPs are also adjacent to unidentified polymorphisms. Consequently, at least in this cell line, sites of strong ASM are almost exclusively attributable to neighboring SNPs. Of the 7,569 sites displaying significant evidence of ASM, only 0.24% were within 10 kb of an imprinted locus (18 sites within 10 kb of 13 distinct imprinted loci), 73 times less than the proportion of ASHM sites within the same distance of an imprinted region (*P* < 2.2 × 10^−16^, χ² test). Examining the proportion of these ASM sites within 10 kb of a known site of ASE showed that only 1.84% of sites were within this distance, which is also significantly lower than the proportion of ASHM sites (19.6%, as discussed above, *P* < 2.2 × 10^−16^, χ² test).

Only two known imprinted regions displayed significant evidence of both ASM and ASHM in this analysis: the region around the *GNAS1* gene on chromosome 20 and the imprinted locus on 15q11-13. Whereas many sites of ASM are found all along chromosome 15, no sites of significant ASHM are found on this chromosome outside this known imprinted locus. These results show that, at least in hESCs, sites of ASHM are better than sites of ASM as markers of imprinted regions, and that ASHM is consequently likely to be more useful in any search for novel imprinted loci.

### Allele-specific histone modifications are enriched at disease-causing micro-indel regions

Regions of allele-specific chromatin and gene inactivation are likely to be particularly susceptible to functional perturbations following deletions. Deletion of a single functional allele at such regions is more likely to be deleterious than a similar single deletion where two copies of a region are functional and one remains after the deletion event. We therefore examined whether sites of ASHM were located in close proximity to loci at which deletions have been implicated in the onset of developmental disorders [36]. We focused on developmental disorders because of the likely importance of embryonic stem cells in their progression. In total, nine sites of ASHM (17.6%) were found to be within 250 kb of four distinct regions known to be deleted in a developmental disease or syndrome (see Additional file 3), corresponding to a modest but significant enrichment over the proportion of all high-coverage sites within the same distance (6.7% of background sites, *P* = 0.004, χ² test; significant enrichments were also seen at 500 kb and 1 Mb). To further narrow this list to the strongest candidates at which ASHM is likely to be associated with a disease state, we sought examples of those loci for which there was also evidence for allele-specific expression within close proximity (site of ASE with *P* < 1 × 10^−7^ within 10 kb). After this further filtering, seven sites remained; five at the known imprinted region on chromosome 15q11-13, and two at a second region located on 17q21.31.

The 15q11-13 region is a well-characterized imprinted locus, and imprinting at this region has been shown to be associated with a variety of disorders including PWS, Angelman syndrome (AS), and autism spectrum disorders [37,38]. This region displays clear evidence of ASHM, ASE, and ASM (Figure 2). Detection of ASHM at this locus is reassuring, being one of the few previously known locations of ASHM. ASHM at this locus is largely driven by enrichment of the H3K9me3 histone modification, a marker of transcriptional repression, along a single haplotype (Figure 3).

The region at 17q21.31 has previously been shown to contain two haplotypes, H1 and H2 (the latter inverted relative to H1), and our analysis of haplotype marking variants showed that the H1 cell line is heterozygous for these haplotypes. One of the key genes in this region is *microtubule-associated protein tau* ( *MAPT*), which is known to encode the protein that forms neurofibrillary tangles in the brains of people with Alzheimer’s disease [39]. A number of studies have found associations between the H1 haplotype and neurodegenerative disorders such as Parkinson’s disease [39], and therefore it has been assumed that MAPT underlies these disorders. Although not expressed in embryonic stem cells, MAPT has previously been shown to be preferentially expressed from the H1 haplotype, although the mechanism behind this is unknown [40]. This region shows copy number variation in some Europeans (see Methods); however, the SNPs we used for analysis in this region could be unambiguously phased with the haplotype marking variants, and we also found further genes flanking *MAPT* to be preferentially expressed from the H1 haplotype. Our analysis of the ASHM sites at this locus (17:41700635 and 17:41700765) showed that the H1 haplotype is preferentially associated with the H3K4me3 histone modification linked with gene activation, whereas the H2 haplotype is preferentially associated with the repressive H3K27me3 marker, suggesting that there is an association between the haplotype-specific patterns of ASHM at this locus and the observed patterns of ASE.

The presence of ASHM at these two disease loci is unlikely to be due to chance; of all the sites of high ChIP sequencing coverage in our dataset, only 0.5% (95% confidence interval 0.45 to 0.57%) were within close proximity to a locus showing ASE that is also a known locus of microindels leading to disease. Therefore, assuming independence between sites of ASHM and disease-causing microindels, of the 51 sites displaying evidence of ASHM, we would expect at most one of these to be in close proximity of such a locus by chance. However, of the sites displaying evidence of ASHM, seven sites (13.7%) were in fact in close proximity to such regions (27.4-fold enrichment; *P* < 2.2 × 10^−16^, χ² test) and all of these sites were either at the 15q11-13 or 17q21.31 loci.

We also found persuasive evidence of allele-specific expression at a third locus showing both ASHM and microdeletions associated with the onset of developmental disorders; however, the ASE *P*-value at this location at 17q12 was slightly below our stringent cut-off point ( *P* = 3.8 × 10^−6^). Nevertheless, all 19 RNA sequencing reads mapping to a directly genotyped SNP at this locus specifically carried one allele, suggesting that only a lack of sequencing depth led it to fail the strict ASE significance cut-off. We found that the site of ASHM maps to a putative regulatory region bound by a variety of transcription factors, and that the ASE corresponds to an unannotated, spliced transcript directly adjacent to this locus (see Additional file 4). The H3K27me3 repressive marker is strongly associated with one allele at this locus, with the active H3K36me3 modification to a lesser extent preferentially associated with the second, supporting the hypothesis that this site of ASHM is potentially associated with the observed ASE.

### Overlap of allele-specific histone modification between cell types

If the enrichment at sites of ASHM around loci known to be deleted in developmental disorders is indeed specific to the H1 cell line as a result of its importance in development, we would not expect to see a similar enrichment in a differentiated cell line; however, if this enrichment was an artifact of these regions being present in variable copies in the human genome, we would expect to see such enrichments.

Thus, we applied our protocol to a second, fetal lung fibroblast-derived cell line (IMR90), and found 16 sites of ASHM (see Additional file 2), of which none were within 250 kb of a locus known to be deleted in a developmental disease or syndrome (as opposed to the 9/51 sites in the H1 cell line). This tentatively supports our hypothesis that the sites of ASHM identified in the H1 embryonic stem cell line are indeed specifically associated with these regions; however, because of the small number of ASHM sites seen in IMR90, additional data and further analysis are required.

None of the 16 sites of ASHM detected in the IMR90 cell line was present in the H1 cell line. Only a subset of relevant heterozygote sites were though present in both datasets, and the sequencing coverage differed between the cell lines for each modification and at different positions in the genome. Closer examination of the results from both cell lines did not provide any evidence that there was a substantial enrichment of sites of ASHM in the two cell lines located in close proximity. However, it cannot be excluded that there were some sites of ASHM present in both samples, but that there was insufficient power to detect them in one or both datasets. For example, the nearest heterozygote site in the IMR90 dataset to the sites of ASHM in the H1 cell line (Figure 3) had a suggestive ASHM *P* value of 4.7 × 10^−4^. This *P*-value is largely attributable to the preferential association of the H3K9me3 histone marker with one allele at this locus, as also observed in the H1 cell line. It is therefore likely that similar ASHM is present in both cell lines at this locus, but there is insufficient coverage of this region in the IMR90 cell line to obtain a *P*-value below the strict threshold for genome-wide significance we used in this study.

## Conclusion

To date, there has been little investigation of the extent and nature of ASHM across the human genome. In this study, we undertook a comprehensive, genome-wide investigation of ASHM in an hESC line. Of more than 1.7 million putative heterozygote sites identified in this study, more than 81,000 displayed high ChIP-sequencing coverage (35*x* or more, see Methods), of which 51 displayed significant evidence of different allele ratios in the reads from two or more histone-modification datasets. The results in this study therefore highlight that although sites of ASHM are found throughout the genome, they are probably not common. Owing to this being the first genome-wide analysis of ASHM in humans, a relatively stringent approach was used in this study, which probably resulted in a higher false-negative rate. However, the data used were combined from two large international sequencing projects, so although the generation of more data and for more modifications would probably identify further sites, we can still state that ASHM does not seem to be widespread, at least in hESCs. In yeast, it has been shown that single base changes can lead to predictable changes in nucleosome positioning [41]; however, the results from the current study argue against SNPs having a substantial widespread effect on the modifications each nucleosome carries. Changes in the underlying DNA are rarely associated with changes in the modification carried by any overlying nucleosome. This is in broad agreement with a recent study that could find no evidence for selection acting on particular base changes at DNA underlying nucleosomes carrying different histone modifications [42]. The fact that there is little evidence that underlying DNA has a substantial affect on histone modifications is perhaps unsurprising; patterns and distributions of histone modifications vary between cell types even when from the same genetic background. Histone modifications do not seem to be strongly reliant on the DNA sequences immediately underlying the nucleosomes on which they occur. These data are in contrast to the results we obtained when investigating ASM, which was found to be relatively common and closely associated with the underlying DNA, with almost all sites of ASM identified being attributable to a neighboring SNP. These results would suggest there are probably different mechanisms controlling ASHM and ASM in embryonic stem cells, and that one does not necessarily lead to the other.

We have shown in this study that despite sites of ASHM not being widespread, they are likely to be important. ASHM sites were found to be enriched around genes displaying evidence of ASE. Although this is not evidence of a direct link, it is likely, given the known role of histone modifications in gene expression, that at least some of these sites of ASHM are directly associated with allele-specific gene expression. ASM, which is already known to be directly associated with allele-specific gene expression, was substantially less enriched around sites of ASE. Consequently, the few sites of ASHM identified in this study would seem to be having a disproportionate affect on gene expression.

Sites of ASHM were also substantially more enriched around known imprinted genes than sites of ASM. We found that 1 in 6 sites of ASHM were within 10 kb of a known imprinted locus, as opposed to 1 in 417 sites of ASM. This suggests that ASHM is a substantially stronger marker of imprinted genes in embryonic stem cells than is ASM, and will probably be more informative in any searches for novel imprinted loci. It would seem that the reason that sites of ASM display such limited enrichment around imprinted regions is because the most significant sites are overwhelmingly the result of adjacent polymorphisms. This result also suggests that strong epialleles (DNA methylation differences between alleles that are not attributable to underlying DNA polymorphisms) are likely to be relatively rare in hESCs. The lack of strong enrichment of sites of ASM around imprinted regions may also be partly a result of imprinted regions having smaller differences in methylation between chromosomes across longer stretches, compared with sites at which CpG motifs are disrupted by a heterozygous SNP. Smaller differences in methylation levels will be difficult to detect without extremely high coverage, when robustly accounting for the substantial number of sites tested genome-wide.

Interestingly, despite the highly significant enrichment of ASHM sites around known imprinted regions, most ASHM sites were not associated with such regions. This suggests that either there are a substantial number of imprinted regions yet to be defined in the human genome or that ASHM is often located distinct from imprinted loci.

Analysis of the distribution of ASHM sites relative to the position of known microdeletion syndrome loci led to the discovery that one in six sites of allele-specific histone modification were within 250 kb of a locus at which deletions have been associated with developmental disorders. The observation of sites of ASHM at the PWS genomic interval on 15q11-13 is in agreement with previous studies [43]. Targeted studies in mice looking at the patterns of histone modification at this imprinted locus in murine embryonic brain samples have identified enrichment of H3K4me2 and H3Ac modifications along the paternal chromosome, with H4K20me3-, H3K36me3-, H3K9me3-, and H3K79me3-modified nucleosomes enriched along the silenced maternal copy [43]. Our observation of preferential enrichment of the H3K9me3 marker along the same phased haplotype in this study is therefore in broad agreement with the observed parent-of-origin chromosome bias of this modification in mice. However, the observed bias of the H3K36me3 histone modification along the silenced maternal chromosome in the same mouse study was more surprising, given that this histone mark has previously been associated with active, transcribed regions [44]. In the current study looking at hESCs, we found sites in this region preferentially covered by nucleosomes carrying H3K36me3 were located on both chromosomes, suggesting that this modification is not preferentially associated with either chromosome copy in hESCs. This observation of sites of H3K36me3 on both the silenced and active copies of this region in humans is in agreement with the broader observation that this marker is found at both transcriptionally active and repressed regions in mice, including mouse embryonic stem cells [43].

Although rare, ASHM is consequently likely to play an important role in human development. The location of sites of ASHM at key imprinted and ASE loci and at sites associated with developmental disorders suggests that ASHM is an important but as yet undercharacterized phenomenon involved in embryonic development.

## Methods

### Heterozygote SNP imputation analysis

The genotypes at 480,621 SNP positions in the H1 cell line were obtained from the report of Mosher *et al*. [20], and used in conjunction with the IMPUTE program [23] to determine the putative positions of heterozygote SNPs and haplotypes in this cell line. Imputation was carried out using the combined HapMap 3 (release 2) and 1000 Genomes pilot CEU (Utah residents with Northern and Western European ancestry) haplotypes available at the IMPUTE website (H1 has previously been shown to be of European origin [20]). All SNPs present on the genotyping array were given a predicted probability of 1 for the corresponding genotype. In total, 1,728,322 positions had a predicted probability of greater than or equal to 0.5 of being a heterozygote, that is, were more likely to be a heterozygote than a homozygote. This number is in broad agreement with the expected number of heterozygote sites in an individual [21]. However, as this set of polymorphic sites was likely to contain a large number of false positives, all of these putative heterozygotes were also validated using whole-genome Bisulfite sequencing data as detailed below.

### Detection of allele-specific DNA methylation

Bisulfite sequencing reads were mapped to the hg18 version of the human reference genome using the Bismark software [35]. Seven libraries were excluded from subsequent analysis because they gave substantially different estimates of the number of methylated sites in the H1 genome than the other 200 libraries (see Additional file 5 for more details). In total, the mean coverage of mapped reads across both strands was 32*x*. The reads carrying each allele at each imputed heterozygote site was determined for reads mapping to the forward and reverse strand independently (sites of C/T polymorphisms on the respective strand were excluded).

Analysis of the Bisulfite sequencing data showed that it could successfully be used to confidently identify heterozygote sites in the H1 cell line. Filtering positions according to known sites of polymorphisms in the 1000 Genome and HapMap datasets, and with at least two reads being present in the Bisulfite sequencing data carrying each known allele, allowed us to capture heterozygote sites with a very low false-positive rate. Comparison of these SNP calls to the genotyping-array data highlighted that the sensitivity and specificity of detecting heterozygotes using this approach were 78.5% and 99.7% respectively (positive predictive value of 99.2%), that is, the false-positive rate was 0.3%. Consequently, this Bisulfite sequencing data was used to further filter the putative heterozygote sites identified in the imputation analysis. Only sites with a heterozygote probability of ≥0.5 from the imputation analysis, and with at least two reads for each of the two respective alleles seen at the corresponding position in the Bisulfite sequencing data, were retained for downstream analyses (including the identification of ASHM). This approach ensured that the false-positive rate in this study was as low as possible. After this filtering, 1,230,096 heterozygote sites were left. In the absence of genotyping data for IMR90, we also used the Bisulfite sequencing data to identify putative heterozygote sites as described.

To detect sites of ASM, the methylation status at each position along each mapped Bisulfite sequencing read was determined (excluding known polymorphic positions in the 1000 Genomes and HapMap data from IMPUTE), and the total number of reads displaying evidence of methylation at a position and carrying a given allele at the respective heterozygote site was calculated. Bases with Phred-scaled qualities of less than 13 were excluded from these calculations. The proportions of methylated and unmethylated bases at a given position were then compared between groups of reads carrying each allele at the corresponding heterozygote site using a Fisher’s exact test. The cut-off for significance in this analysis was set to 1 × 10^−8^, which approximately corresponds to a Bonferroni-corrected *P*-value of 0.05 (3,410,426 putative sites of ASM tested). It should be said that a less stringent threshold could have potentially been used, not least because by applying a Bonferroni correction, we assumed that methylation levels at neighboring sites are independent, but this is known not to be the case [1]. The threshold used in this study can therefore be considered robust.

### Detection of allele-specific histone modifications

All sequence data used in this project were generated by the ENCODE [24,45] or National Institute of Health Roadmap Epigenomics [25] projects (for a full list of the datasets used see Additional file 6). All ChIP-sequencing reads were mapped to the hg18 build of the Human Reference Genome, using the Bowtie short read aligner with the ‘−best’ flag [46]. In total 1,389,697,934 reads were successfully mapped (an average of 60.4 million per histone modification; see Additional file 1). The numbers of reads carrying each possible allele (with respect to the positive strand) at all of the imputed heterozygous positions (after filtering using the Bisulfite sequencing data as described above) were determined for each histone modification. Low-quality read bases (Phred-scaled quality < 13) were ignored. A 4 × 23 contingency table was constructed for each site, corresponding to the number of reads for each allele and each modification. Having removed columns and rows that summed to 0, this table was used as the input of a Fisher’s exact test to identify sites of ASHM (see Additional file 7 for further details of this approach). A significance threshold of 1 × 10^−7^ was used in this analysis. Owing to the probably higher false-positive rate in the previous 1000 genomes dataset, sites not in the later (October 2011) 1000 Genomes release were ignored. Under conservative assumptions that the mean power to detect sites of ASHM in this study was on average around 0.1, and that only 0.001% of the approximately 15 million nucleosomes expected to be found along the human genome in fact display evidence of ASHM, the posterior odds will be 10 to 1 in favor of any site exceeding this threshold being a true association [47].

This test identified sites at which the proportion of reads carrying a particular allele was different between at least two histone-modification datasets. Although the imputation analysis and subsequent filtering according to the Bisulfite sequencing data would probably have produced a number of false-positive heterozygotes, this approach meant that they could not be detected as sites of ASHM. A putatively heterozygous position requires a substantial difference in the proportions of reads carrying each allele between datasets to be called as a location of ASHM. At true homozygote positions, only one allele will be identified (sequencing errors are not expected to produce a sufficient number of alternative alleles to reach significance, and should also occur at relatively uniform rates between datasets). This approach also ensured that the putative sites of ASHM were not in fact a result of differences in nucleosome occupancy between chromosomes. Such differences may lead to different observed proportions of reads carrying each allele within a particular modification dataset overlapping a site, but should not change the proportion of reads carrying each allele between modification datasets (see Additional file 7 for a fuller explanation of the method for calling sites of ASHM).

Despite the resulting low SNP false-positive rate, it is possible that differences between duplicated regions in the human genome could be mistaken as heterozygote polymorphisms. Although our approach of only identifying sites at which the allele ratios are different between at least two histone-modification datasets would exclude many potential false positives arising from such duplication events (as duplication events in which the modification state has stayed the same or has been completely lost will not be detected as sites of ASHM), differences between the histone-modification spectrums at duplicated sites could potentially be erroneously called as sites of ASHM. For example, although large (>3 to 400 kb) duplications are incredibly rare at the sites associated with developmental delay used in this study [36], smaller sections of the 17q21.31 region have previously been shown to be duplicated in some Europeans, and to have higher coverage in corresponding sequencing datasets [48]. To investigate whether duplication events might underlie the ASHM sites identified in this study we therefore first investigated whether there was evidence of an excess of Bisulfite sequencing read depth at these positions (the Bisulfite data being used because it was untargeted whole-genome sequencing data). Sites of duplications have been shown to lead to an increase in coverage of the respective sites [48]; however, we found that the mean coverage at the ASHM sites in this study was is in fact lower than the mean coverage across the whole genome (see Additional file 8), suggesting that as a group they are not enriched with duplicated sites. Using the 1000 Genomes CEU data release of October 2011, we also ensured all the SNPs underlying sites of ASHM were in Hardy-Weinberg equilibrium (HWE), because duplications and high-frequency copy number variations (such as that at 17q21.31) would be expected to cause false-positive SNPs to deviate from HWE [49]. Although the distribution of HWE values was similar for both the ASHM and all high-coverage sites (see Additional file 8), suggesting that there is no systematic bias at ASHM sites towards SNPs out of HWE, we excluded three sites whose HWE *P*-value was less than 0.05. This is probably over-conservative, given that we would in fact expect the same number of SNPs to have a HWE *P*-value lower than this cut-off value, purely by chance. The same filtering was applied to the IMR90 sites of ASHM. None was out of HWE, and there was no substantial systematic bias in read-depth coverage. Therefore, although it is not possible to say unequivocally that every ASHM site identified in this study has only two copies in these cell lines, there is no evidence that the ASHM sites identified are, as a group, false positives resulting from duplication events.

In order to allow clustering of sites displaying significant evidence of ASHM by their histone-modification patterns, we calculated the degree of allelic imbalance for each individual modification at each site using a binomial test. Each of the resulting *P*-values for each modification at each site were then log-transformed, and sites clustered by their squared Pearson’s correlation coefficient using the *R* statistical package [50]. The six clusters were identified by defining a cut-off point in the dendrogram shown in Figure 1A. This cut-off point was chosen by progressively lowering the cut-off until further lowering it resulted in multiple clusters driven primarily by the same modification(s). The modifications driving each cluster were identified by determining those modifications with small binomial *P*-values across all of the sites in the cluster.

To investigate the enrichment of ASHM in and around various annotations, sites of significant ASHM were compared only to those heterozygote sites with high ChIP-sequencing coverage; that is, only those heterozygote sites identified using the imputation and Bisulfite sequencing data that were covered by at least 35 ChIP-sequencing reads (with only sites in the October 2011 1000 Genome release being again retained). A read number of 35 was chosen as the cut-off point as this was the minimum number of reads identified at the 51 sites of ASHM. Comparing sites of ASHM with the 81,029 such sites of high ChIP-sequencing read depth, rather than with all heterozygote positions, consequently ensured that any enrichment at ASHM sites was measured relative to all heterozygote positions covered by modified nucleosomes and at which ASHM was potentially detectable if it existed. The locations of 71 human imprinted loci were obtained from the study of Morrison *et al*. [31] (the PWS/AS locus was treated as one distinct region in this analysis). Sites of deletions associated with developmental delay were obtained from the data of [Cooper *et al*. 36]. SNPs were phased using IMPUTE in conjunction with the 1000 Genomes CEU haplotypes [21].

### Detection of allele-specific expression and *Pol*II binding

All RNA-sequencing and *Pol*II ChIP-sequencing reads were initially mapped using Bowtie with the ‘–best’ flag [46]. For details of the datasets used see Additional file 6. Those RNA-sequencing reads that were unmapped by Bowtie were then run through the spliced-read mapper TopHat [51]. Sites of ASE and *Pol*II binding were called by determining the number of reads at each heterozygote site carrying each allele, and deviations from the expected 50/50 ratio were assessed using a binomial test. Reads in which the base at the heterozygote site had a Phred-scaled quality of less than 13 were excluded from this analysis. Owing to the high sensitivity for heterozygote false positives in this analysis (all such false positives would probably be called as highly significant sites of ASE) we used the Bisulfite sequencing data to minimize the false positive rate as previously described, that is, only SNP genotyping-array heterozygote SNPs or imputed sites with a heterozygote probability greater than 0.5 and at least two Bisulfite sequencing reads carrying each allele were kept. A *P*-value threshold of 1 × 10^−7^ was used in this analysis.

### Haplotype analysis

SNPs were phased using IMPUTE in conjunction with the 1000 Genome CEU haplotypes. In total, 19 inversion-marking SNPs with a dbSNP rs ID taken from the study of [Donnelly *et al*. 52] were used to determine which phased haplotype was H1 and which H2 at the 17q21.31 locus (although there are 21 inversion-marking SNPs listed in this paper, 1 did not have an rs ID so was not imputed, and the listed alleles for another did not match those found in the 1000 Genomes data, and was therefore excluded). All 19 SNPs agreed in the discrimination of the H1 and H2 haplotypes.

## Abbreviations

AS, Angelman syndrome; ASHM, Allele-specific histone modification; ASM, Allele-specific methylation; ASE, Allele-specific expression; ChIP, Chromatin immunoprecipitation; GWAS, Genome-wide association studies; hESC, Human embryonic stem cell; HWE, Hardy-Weinberg equilibrium; PWS, Prader-Willi syndrome; SNP, Single-nucleotide polymorphism.

## Competing interests

The author(s) declare that they have no competing interests.

## Authors’ contributions

JGDP and CAMS conceived of the study and drafted the initial manuscript. JGDP and PT undertook the bioinformatic analysis. All authors contributed to and approved the final manuscript.

## Supplementary Material

Additional file 1 Number of mapped reads for each dataset used in this study. Click here for file

Additional file 2 Read counts for each allele and each histone modification at the sites of ASHM identified in this study. Click here for file

Additional file 3 Sites of ASHM and their distance to corresponding annotations. Click here for file

Additional file 4 Location of ASE and ASHM sites at the 17q12 locus. Click here for file

Additional file 5 H1 Bisulfite sequencing dataset comparison Click here for file

Additional file 6 Datasets used in this analysis. Click here for file

Additional file 7 Description of method for calling sites of ASHM. Click here for file

Additional file 8 ASHM site quality control (A) Coverage of Bisulfite sequencing reads across the whole genome and at sites of ASHM only. 0.26% of sites in the whole genome analysis had coverages of greater than 100, but these have been excluded from this plot. The mean coverage at sites of ASHM was 23*x* as opposed to the genome-wide mean coverage of 32 *x*. (B) Hardy-Weinberg equilibrium (HWE) *P*-values at 54 ASHM site polymorphisms (i.e. before HWE filtering) and at all high-coverage sites. Click here for file
