# Supplementary figures and images for: A genome-wide screen in human embryonic stem cells reveals novel sites of allele-specific histone modification associated with known disease loci
Source: Epigenetics Chromatin. 2012 May 19;5:6. doi: 10.1186/1756-8935-5-6 (PMC3438052; doi:10.1186/1756-8935-5-6)

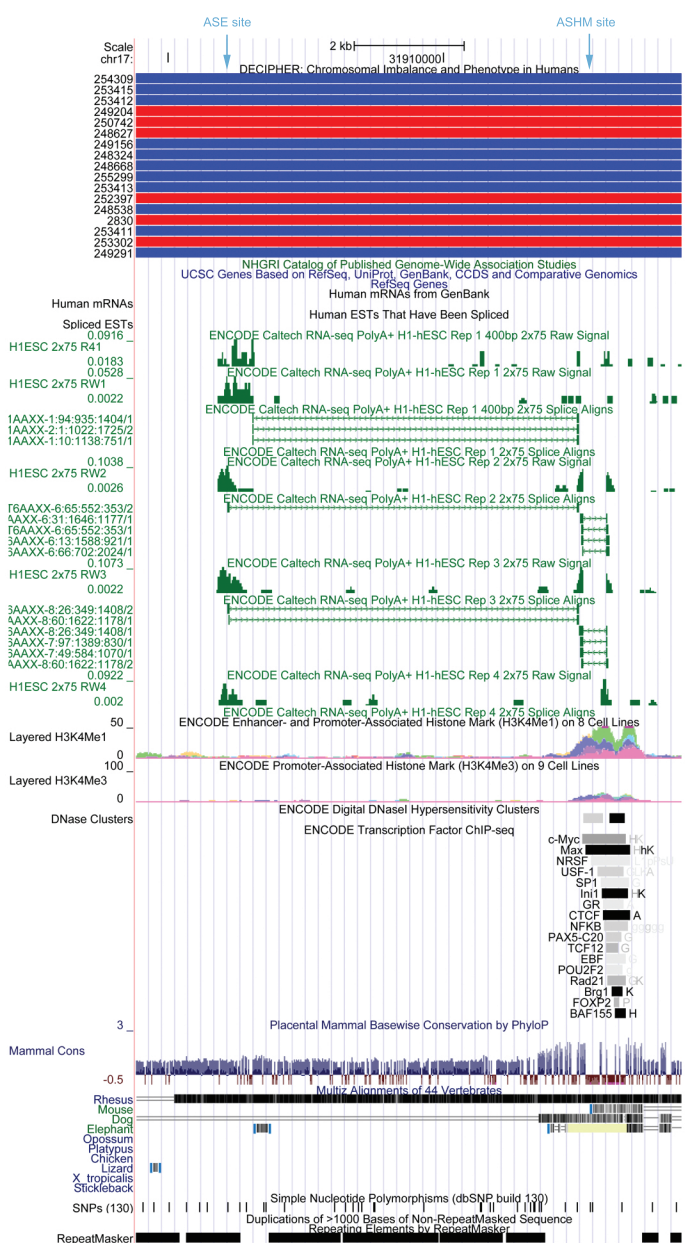

Supplement: Additional file 4 — Location of ASE and ASHM sites at the 17q12 locus. [file 1756-8935-5-6-S4.pdf]

A

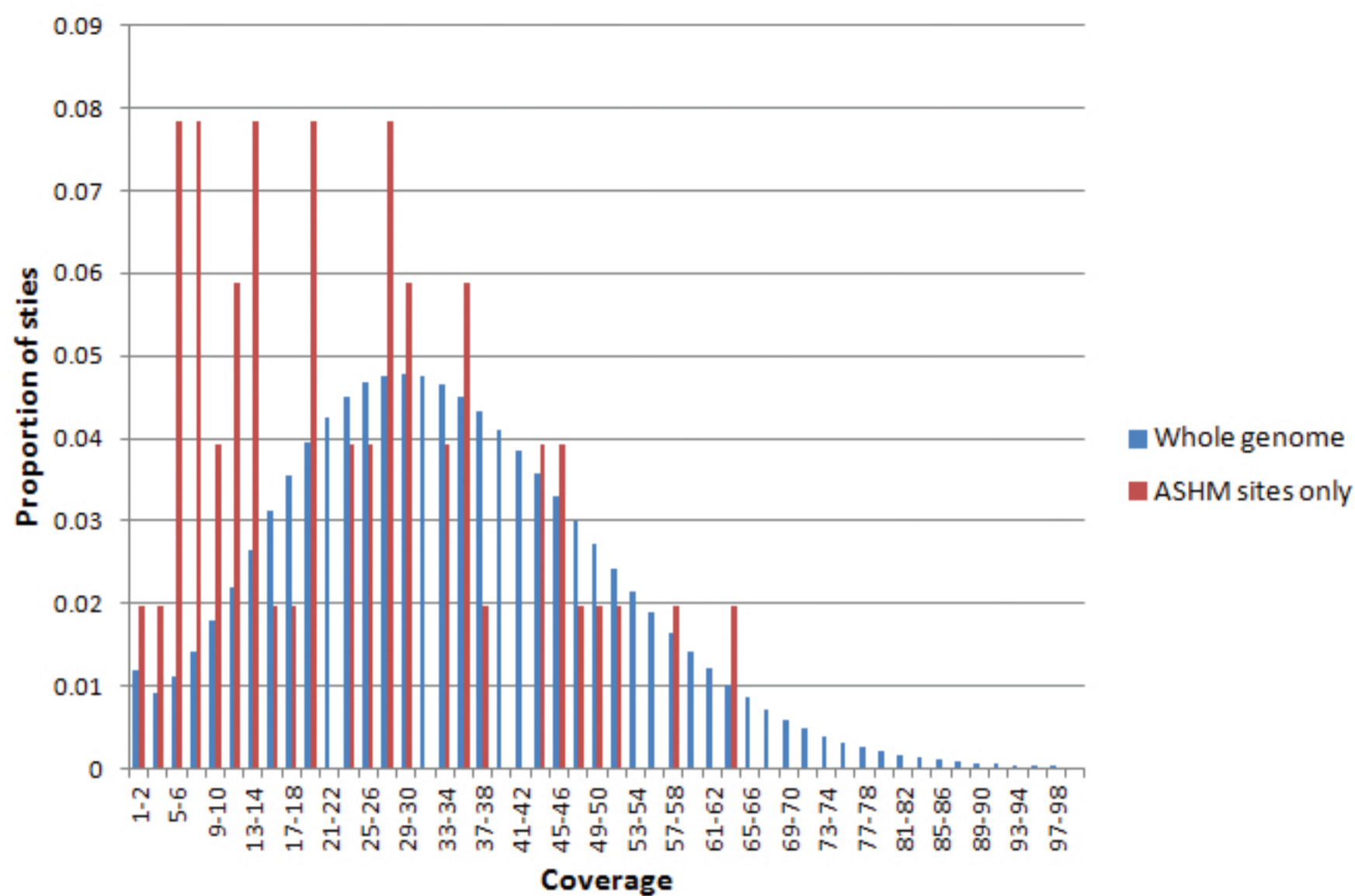

B

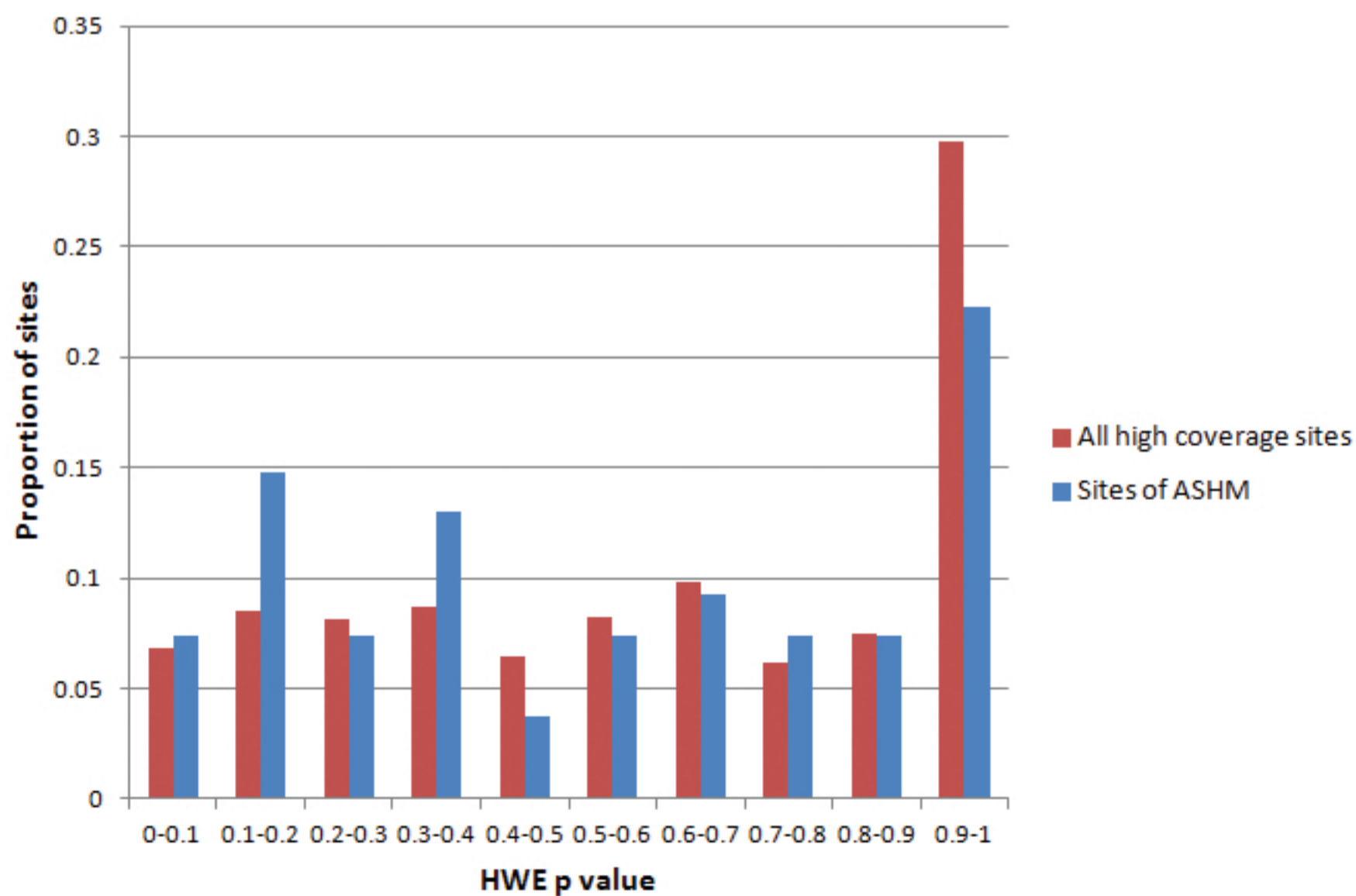

Supplement: Additional file 8 — ASHM site quality control (A) Coverage of Bisulfite sequencing reads across the whole genome and at sites of ASHM only. 0.26% of sites in the whole genome analysis had coverages of greater than 100, but these have been excluded from this plot. The mean coverage at sites of ASHM was 23x as opposed to the genome-wide mean coverage of 32 x. (B) Hardy-Weinberg equilibrium (HWE) P-values at 54 ASHM site polymorphisms (i.e. before HWE filtering) and at all high-coverage sites. [file 1756-8935-5-6-S8.pdf]
