# Supplementary material for: A genome-wide screen in human embryonic stem cells reveals novel sites of allele-specific histone modification associated with known disease loci
Source: Epigenetics Chromatin. 2012 May 19;5:6. doi: 10.1186/1756-8935-5-6 (PMC3438052; doi:10.1186/1756-8935-5-6)
Supplement: Additional file 7 — Description of method for calling sites of ASHM. [file 1756-8935-5-6-S7.pdf]

**A**

| | Allele A | Allele B | Allele ratio |
|---|---|---|---|
| **Modification 1** | 45 | 45 | 1:1 |
| **Modification 2** | 6 | 6 | 1:1 |
| **Modification 3** | 120 | 120 | 1:1 |

p = 1

**B**

| | Allele A | Allele B | Allele ratio |
|---|---|---|---|
| **Modification 1** | 45 | 45 | 1:1 |
| **Modification 2** | 6 | 60 | 1:10 |
| **Modification 3** | 120 | 120 | 1:1 |

p = 4.684e-10

**C**

| | Allele A | Allele B | Allele ratio |
|---|---|---|---|
| **Modification 1** | 60 | 30 | 2:1 |
| **Modification 2** | 8 | 4 | 2:1 |
| **Modification 3** | 160 | 80 | 2:1 |

p = 1

**D**

| | Allele A | Allele B | Allele ratio |
|---|---|---|---|
| **Modification 1** | 60 | 30 | 2:1 |
| **Modification 2** | 8 | 4 | 2:1 |
| **Modification 3** | 80 | 160 | 1:2 |

p = 4.924e-08

For each site of potential ASHM a 4x23 contingency table was constructed (with rows or columns that summed to 0, i.e. modifications or alleles with no corresponding reads, being removed). These tables contained the number of reads carrying each of the four possible bases at the corresponding position for each of the 23 histone modifications examined. Each contingency table was used as the input to a Fisher's exact test and consequently, at its simplest, this test identified those sites where at least one modification displayed an unusual allele ratio. Figures A to D above help to explain this approach further. To simplify these examples the contingency tables have been restricted to 2 alleles and 3 modifications. The basic assumption underlying any investigation of allele-specific phenomena is that the allele ratio is expected to be 1:1 at the majority of sites, i.e. most sites show no allelic bias. Figure A shows an example of this situation; although there may be different numbers of reads for each modification at a site the expectation is that the proportion of reads carrying each allele at a heterozygote site will be approximately equal. As would be expected the Fisher's exact p value corresponding to Figure A is 1, as there is no allelic imbalance at any modification. Figure B on the other hand shows an example where modification 2 is biased towards allele B and consequently a significant p value is obtained for this site, indicating ASHM. However it is possible that imbalances in histone modification allele ratios can happen at a site for reasons other than ASHM. For example there may be biases in the mapping of reads to the reference allele, shifts in nucleosome positioning between chromosomes etc. These systematic biases would be expected to lead to deviations from equality in the allele ratios observed for all modifications, as shown in Figure C. If allelic imbalance at modification 3 in this example (Figure C) was tested in isolation, for example by using a binomial test, this modification would be incorrectly identified as a site of ASHM. However, by testing all modifications together this site is not identified as a location of ASHM, reflected in a Fisher's exact p value of 1, as no modifications display an unusual allele ratio in the context of this particular site (i.e. relative to the other modifications). Figure D shows the same site but where modification 3 now displays an unusual allele ratio relative to the other modifications and is therefore identified as a site of ASHM using a Fisher's exact test. It is worth noting that our approach is therefore potentially conservative, as sites where all 23 modifications show exactly the same allele bias will not be detected as sites of ASHM. However, the approach does ensure that the false positive rate is low and also importantly means substantially fewer tests are performed than if each modification at each site was tested independently, leading to a higher false positive rate or the requirement of a substantially more stringent p value cut-off. Two real examples of sites of ASHM are shown below.

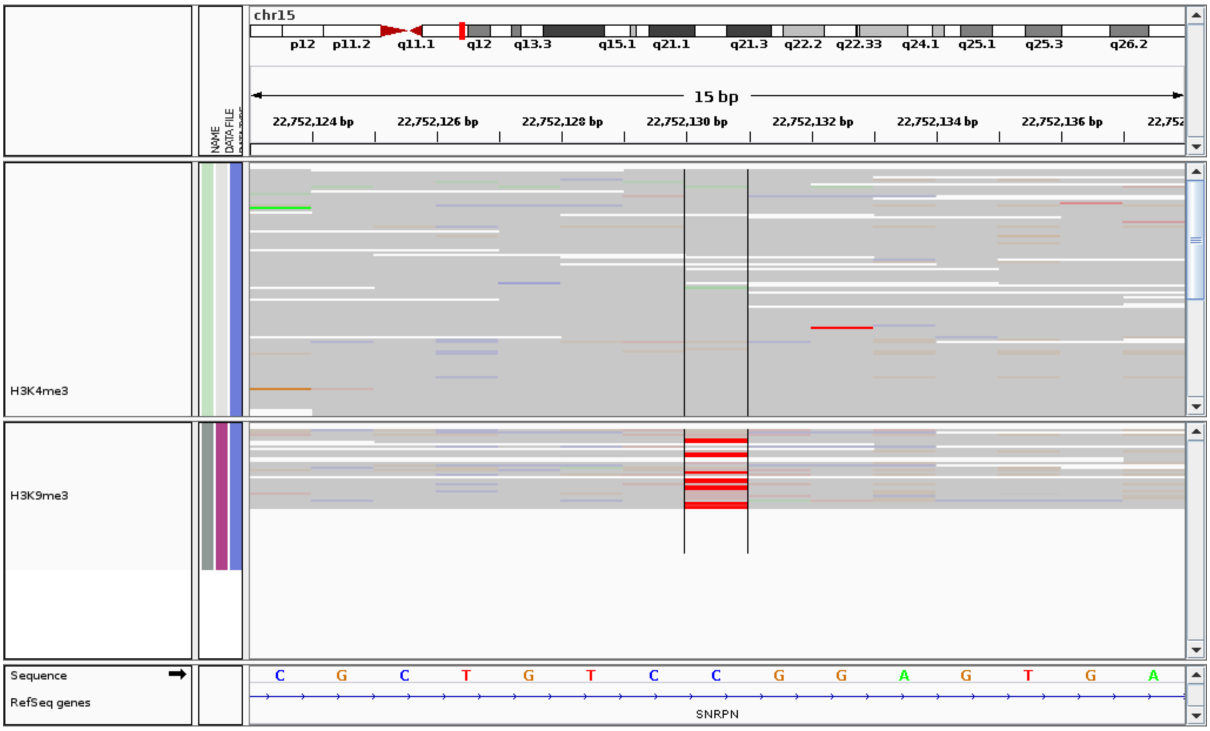


**Figure 1:** Allele imbalances observed at the known imprinted region at chr15:22752130. Although almost all reads corresponding to the H3K4me3 modification carry the reference base at this position (C, represented by grey lines) a large proportion of the reads from the H3K9me3 dataset carry a T at this base (represented by red lines).

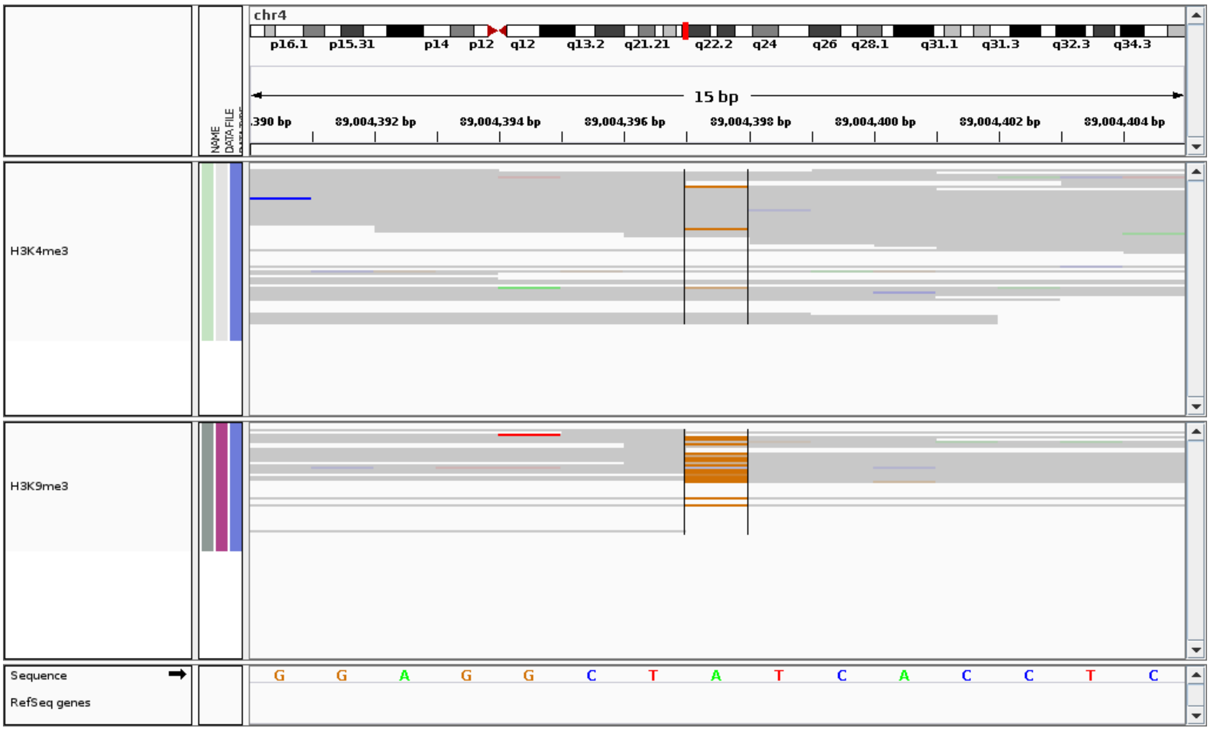


**Figure 2:** A similar pattern of allelic imbalance observed at chr4:89004397 (not known to correspond to an imprinted region). The majority of reads corresponding to the H3K4me3 modification again carry the reference base at this position (A) however the majority of the reads from the H3K9me3 dataset carry a G (orange lines) at this base.
